# Roles of Endothelial Motilin Receptor and Its Signal Transduction Pathway in Motilin-Induced Left Gastric Artery Relaxation in Dogs

**DOI:** 10.3389/fphys.2021.770430

**Published:** 2021-10-28

**Authors:** HongYu Li, LanLan Yang, Ying Jin, ChunXiang Jin

**Affiliations:** ^1^Department of Ultrasound, China-Japan Union Hospital of Jilin University, Changchun, China; ^2^Department of Ultrasound, The First Hospital of Jilin University, Changchun, China; ^3^Department of Hepatopancreatobiliary Medicine, The Second Hospital of Jilin University, Changchun, China; ^4^Department of Breast Surgery, The First Hospital of Jilin University, Changchun, China

**Keywords:** endothelial motilin receptor, signal pathway, nitric oxide, vasorelaxation, dog left gastric artery

## Abstract

**Background:** Motilin increases left gastric artery (LGA) blood flow in dogs *via* the endothelial motilin receptor (MLNR). This article investigates the signaling pathways of endothelial MLNR.

**Methods:** Motilin-induced relaxation of LGA rings was assessed using wire myography. Nitric oxide (NO), and cyclic guanosine monophosphate (cGMP) levels were measured using an NO assay kit and cGMP ELISA kit, respectively.

**Results:** Motilin concentration-dependently (EC_50_=9.1±1.2×10^−8^M) relaxed LGA rings precontracted with U46619 (thromboxane A_2_ receptor agonist). GM-109 (MLNR antagonist) significantly inhibited motilin-induced LGA relaxation and the production of NO and cGMP. N-ethylmaleimide (NEM; G-protein antagonist), U73122 [phospholipase C (PLC) inhibitor], and 2-aminoethyl diphenylborinate [2-APB; inositol trisphosphate (IP_3_) blocker] partially or completely blocked vasorelaxation. In contrast, chelerythrine [protein kinase C (PKC) inhibitor] and H89 [protein kinase A (PKA) inhibitor] had no such effect. Low-calcium or calcium-free Krebs solutions also reduced vasorelaxation. N-nitro-L-arginine methyl ester [L-NAME; nitric oxide synthase (NOS) inhibitor] and ODQ [soluble guanylyl cyclase (sGC) inhibitor] completely abolished vasodilation and synthesis of NO and cGMP. Indomethacin (cyclooxygenase inhibitor), 18α-glycyrrhetinic acid [18α-GA; myoendothelial gap junction (MEGJ) inhibitor], and K^+^ channel inhibition through high K^+^ concentrations or tetraethylammonium (TEA-Cl; K_Ca_ channel blocker) partially decreased vasorelaxation, whereas glibenclamide (K_ATP_ channel blocker) had no such effect.

**Conclusion:** The current study suggests that motilin-induced LGA relaxation is dependent on endothelial MLNR through the G protein-PLC-IP_3_ pathway and Ca^2+^ influx. The NOS-NO-sGC-cGMP pathway, prostacyclin, MEGJ, and K^+^ channels (especially K_Ca_) are involved in endothelial-dependent relaxation of vascular smooth muscle (VSM) cells.

## Introduction

Motilin is a 22-amino acid intestinal peptide and an endogenous ligand of the motilin receptor (MLNR). It is cyclically released during the interdigestive period, but this pattern is halted in response to a meal. Motilin induces gastric phase III of the migrating motor complex (MMC III) in fasting humans and dogs (Lee et al., 1983; Ogawa et al., 2011; Deloose et al., 2015; Kitazawa and Kaiya, 2019), and also simultaneously induces a sustained increase in blood flow (≤ 240% resting blood flow) of the left gastric artery (LGA; Jin et al., 2002). The expression of MLNR has recently been found on the membrane of endothelial cells (ECs) in canine gastrointestinal arteries (Yang et al., 2021). However, the signal transduction pathways by which motilin induces vascular smooth muscle (VSM) relaxation is unclear.

Known functional MLNR expression sites include the myenteric plexus (Ohshiro et al., 2008; He et al., 2015) and gastrointestinal smooth muscle (Miller et al., 2000a,b). Imporantly, motilin peptide fragments have a greater affinity to neuronal tissue compared to muscle tissue (Poitras et al., 1996; Miller et al., 2000a,b), and the activation of different downstream effector molecules by the same receptors in different cell types may vary (Dass et al., 2003). MLNR mRNA or protein expression has also been found on tissues outside of the gastrointestinal tract, such as the hypothalamus and medulla oblongata (Suzuki et al., 2012), the thyroid gland and bone marrow (Feighner et al., 1999), and the lacrimal glands (Sadig et al., 2021). Yet, the physiological function of this expression is not fully understood. However, endothelial MLNR is the molecular basis to allow motilin to regulate gastric artery blood flow in dogs under physiological conditions (Jin et al., 2002; Yang et al., 2021). Thus, it is of physiological and pathophysiological significance to study its signal transduction pathway.

Human MLNR belongs to the class I G protein-coupled receptor (GPCR) family (Feighner et al., 1999). The activation of MLNR on rabbit gastrointestinal smooth muscle cells (SMCs) by its agonist causes Ca^2+^ release from intracellular stores *via* the Gq-phospholipase C-inositol trisphosphate (Gq-PLC-IP_3_) pathway (Depoortere and Peeters, 1995). Although the potency for agonists at the dog MLNR is lower than at humans (Leming et al., 2011), the protein sequence of MLNR in dogs is highly homologous to that in humans and rabbits (71 and 72% sequence identity, respectively; Ohshiro et al., 2008), which has only one variant. Therefore, the MLNR-coupled G protein pathway in ECs of the LGA is properly consistent with that in gastrointestinal SMCs.

Motilin-induced LGA relaxation involves cooperation between ECs and SMCs (Yang et al., 2021). Endothelium-dependent relaxation is achieved through a combination of endothelium-derived prostacyclin (PGI_2_), nitric oxide (NO), and endothelium-derived hyperpolarizing factor (EDHF) by different mechanisms (Kukovetz et al., 1979; Carvajal et al., 2000; Félétou, 2016). The NO-soluble guanylyl cyclase (sGC)-cyclic guanosine monophosphate (cGMP) pathway is essential for the control of vascular homeostasis, especially in elastic arteries (Hiroaki et al., 1996; Leloup et al., 2015). The contribution of PGI_2_ may be negligible in different sized blood vessels; however, there is a compensatory upregulation of PG synthesis when NO-mediated regulation is impaired (Sun et al., 2006). In addition, the importance of the hyperpolarizing mechanism increases as the vessel size decreases (Hiroaki et al., 1996). In the mesenteric arteries of dogs, [Leu^13^]motilin-induced vasorelaxation was inhibited by *Nω*-nitro-L-arginine [10^−4^M; inhibitor of nitric oxide synthase (NOS)], which confirmed the participation of endothelial NO, whereas a high dose of GM-109 (10^−4^M; a MLNR antagonist) only slightly decreased the relaxation, which excluded the role of MLNR (Iwai et al., 1998). However, the fact that GM-109 could inhibit motilin-induced relaxation of dog LGA both *in vivo* and *in vitro* (Jin et al., 2002; Yang et al., 2021) and that motilin induced endothelium-dependent relaxation of dog gastrointestinal arteries (Yang et al., 2021) suggests the irreplaceable roles of both endothelial MLNR and relaxation mediators.

The present work focuses on MLNR, G protein-coupled pathways, and endothelialderived relaxation mediators in the relaxation of LGA rings induced by motilin *in vitro* through the use of specific inhibitors or blockers. Furthermore, the NO and cGMP levels in LGA tissues were also examined to elucidate the essential role of the NO system.

## Materials and Methods

### Animals and Tissue Preparation

This study was conducted in accordance with the Guide for the Care and Use of Laboratory Animals of the National Institutes of Health (NIH Publications No. 8023, revised in 1978). The study protocol was approved by the Animal Care and Use Committee of Jilin University (Permit No. 2016301). All efforts were made to minimize the discomfort of experimental animals. Ninety-six adult mongrel dogs of both sexes (age, 1.5–5.0years; weight, 15–30kg) were used. These purpose-bred mixed-breed animals were used by medical students from the General Theory of Surgery course to practice cutting and suturing the great saphenous vein and trachea.

The dogs were anesthetized with intravenous sodium pentobarbital (30mg/kg), and LGAs (1.8–2.2mm in diameter) were isolated and collected. The animals were euthanized, and tissues were immediately washed with ice-cold gassed (95% O_2_ and 5% CO_2_) and modified Krebs–Henseleit bicarbonate buffer (Krebs solution: 118.0mM NaCl, 4.7mM KCl, 2.5mM CaCl_2_, 1.2mM KH_2_PO_4_, 1.2mM MgSO_4_, 25mM NaHCO_3_, and 10mM glucose, pH 7.4; Iwai et al., 1998). Connective tissue and fat were carefully removed under a dissecting microscope (SZ61, Olympus, Japan), while avoiding over-pulling and clamping.

### Reagents

Porcine motilin (Peptide Institute Inc., Osaka, Japan), acetylcholine chloride (ACh; Sigma, Shanghai, China), and ethylene glycol tetra-acetic acid (EGTA; Sigma) were dissolved in distilled water. U46619 (Sigma) was dissolved in 96% ethanol to 0.4mM (stock solution) and diluted with distilled water before use. Concentrations refer to the final concentration of the drugs in the organ bath and are expressed as mol L^−1^ (M). Dimethyl sulfoxide (DMSO, Solarbio, Beijing, China) and ethanol concentrations in the organ bath were<0.4 and 0.1% (v/v), respectively, and caused no changes in vascular tone. Information regarding all inhibitors/blockers used are listed in Table 1.

**Table 1:** Inhibitors/blockers used with their respective mode of action and final concentrations.

Name	Alias or chemical name	Functions	Company source	Dissolution	Incubation time (min)	Concentration (M)
GM-109	Phe-cyclo[Lys-Tyr(3-tBu)-βAla-]·trifluoroacetate	MLNR antagonist	Peptide Institute	Water	15	10^−10^–10^−5^
NEM	N-ethylmaleimide	G protein antagonist	Sigma	Water, protected from light with pH 7.0–7.4	30	3×10^−5^
U73122	–	PLC inhibitor	MCE, Shanghai, China	DMSO	40	10^−5^
2-APB	2-aminoethyl diphenylborinate	IP_3_R and SOCC blocker	Sigma	DMSO	15	3×10^−4^
Chelerythrine	Chelerythrine chloride	PKC inhibitor	MCE	DMSO	30	10^−6^
H89	–	PKA inhibitor	MCE	DMSO	30	5×10^−6^
L-NAME	*N*^[omega]^-nitro-L-arginine methyl ester	NOS inhibitor	Sigma	Water	15	10^−4^
ODQ	–	sGC inhibitor	Sigma	DMSO	15	10^−5^
Indomethacin	–	cyclooxygenase inhibitor	MCE	DMSO	20	10^−5^
18α-GA	18α-glycyrrhetinic acid	MEGJ inhibitor	Sigma	DMSO	30	7.5×10^−5^
TEA-Cl	Tetraethylammonium chloride	K^+^ channel blocker	Yuanye, Shanghai, China	Water	30	10^−2^
Glibenclamide	–	K_ATP_ blocker	MCE	DMSO	30	10^−6^

*DMSO, dimethylsulfoxide; IP_3_R, inositol trisphosphate receptor; MEGJ, myoendothelial gap junction; MLNR, motilin receptor; NOS, nitric oxide synthase; PKA, protein kinase A; PKC, protein kinase C; PLC, phospholipase C; sGC, soluble guanylate cyclase; and SOCC, store-operated Ca^2+^ channel*.

### Isometric Vascular Tone

Each LGA (approximately 18–30mm long) was cut into 6–10 rings (3mm in length). The samples were mounted between two L-shaped stainless-steel hooks (300μm in diameter) in the organ bath of a multi-wire myograph system (DMT620; Danish Myo Technology, Aarhus, Denmark). The organ bath contained 5ml of Krebs solution continuously gassed with 95% O_2_ and 5% CO_2_. The temperature and pH of the buffer were maintained at 37°C and 7.4. Vascular tension was recorded using LabChart Data Acquisition Software (LabChart 8.0; AD Instruments, New South Wales, Australia). The arterial rings were passively stretched to a tension of approximately 15–20mN [the optimal initial tension was determined in previous experiments (data not shown)], maintained under tension for approximately 60min, and washed every 15min. After tension was stabilized, tissue viability was assessed in 60mM KCl (by replacing NaCl with an equimolar amount of KCl in the Krebs solution) before each experiment.

The rings were contracted using U46619 (5×10^−8^M; thromboxane A_2_ analog). Once the U46619-induced tension remained constant, motilin was added to measure its relaxation effect. Only one concentration of motilin was added to each assay to avoid tachyphylaxis (Mitselos et al., 2007). Endothelium-intact rings were incubated with different inhibitors/blockers for 15–40min before treatment with U46619 (Table 1). In the Ca^2+^-free Krebs solution, Ca^2+^ was replaced with 10^−3^M EGTA. Endothelial integrity and endothelial removal were verified using acetylcholine (10^−5^M) at the end of each test, corresponding to a relaxation rate (RR) of >80 or<10%, respectively.

Relaxation rates were expressed as a percentage decrease in the tension induced by U46619 according to the following formula:


$$RR=100\%\times \left(T-L\right)\times T^{-1}$$

For the calculation of the inhibition rate (IR), tension was normalized to the corresponding values of the control group using the formula:


$$IR=100\%\times \left[\left(Tc-Lc\right)-\left(Ti-Li\right)\times \left(Tc/Ti\right)\right]\times {\left(Tc-Lc\right)}^{-1}$$

where *T* is sustained taension, *L* is the lowest tension, *i* represents the inhibitor group, and *c* represents the control group.

Concentration-response curves were analyzed using nonlinear regression analysis with variable slopes in GraphPad Prism version 9 (San Diego, California, United States). The x% effective concentration (EC_x_) and Hill slope were calculated automatically.

### Measurement of NO and cGMP Levels in the LGA

Left gastric artery tissues were collected and homogenized as described previously (Schachter, 2007). Tissues from three dogs were pooled, and the experiment was repeated three times. NO and cGMP levels were measured without any drugs (blank group) or following treatment with motilin (9×10^−8^M; motilin group). In certain experimental groups, tissues were incubated with GM-109 (10^−5^M) and L-NAME (10^−4^ M; for detecting NO and cGMP), and ODQ (10^−5^ M; for measuring cGMP) before treatment with motilin. The acetylcholine (10^−5^M) group was used to confirm the viability of ECs.

Tissues were homogenized in a solution containing 10.0mM Tris-HCl, 0.1mM EDTA-2Na, 10mM sucrose, and 136.7mM NaCl (pH 7.4; weight to volume ratio of 1:9). The homogenate was centrifuged at 2,400×*g* (5,000rpm) for 10min at 4°C, and the supernatant was assayed. Protein concentration was measured with a bicinchoninic acid total protein assay kit (A045-3; Jiancheng, Nanjing, China) using bovine serum albumin as the standard, and the results were expressed as μmol ml^−1^. NO concentrations were determined using a nitric oxide assay kit (S0023; Beyotime, Haimen, China) and expressed as μmol per g of protein. cGMP concentrations were measured using a canine cGMP ELISA kit (Cat No. ela05471Ca, SANCHEZ, Colorado, United States) and expressed as pmol per mg of protein.

### Statistical Analysis

Data were expressed as means±SEM. Changes in RR were analyzed using one-way ANOVA (Brown-Forsythe and Welch ANOVA tests for non-matched and paired data, and Dunnett T3 multiple comparisons test for multiple comparisons between the treatment groups), where n corresponds to the number of dogs. One LGA ring was obtained from each dog for analysis. Changes in RR induced by motilin (at EC_50_) with and without inhibitors were analyzed using a two-tailed Student’s paired *t*-test, where *n* represents the number of dogs. Two LGA rings from each dog were incubated with and without inhibitors, respectively. Changes in RR induced by motilin (at EC_50_) in Krebs solution containing different Ca^2+^ concentrations ([Ca^2+^]) were analyzed using one-way ANOVA (Geisser-Greenhouse correction for matched data, and the Holm-Sidak method for multiple comparisons between the treatment groups), where *n* corresponds to the number of dogs. Four LGA rings from each dog were incubated with 2.5×10^−3^, 1.25×10^−3^, 0.625×10^−3^, and 0M of Ca^2+^, respectively. Changes in the levels of NO and cGMP under different treatment conditions (i.e., motilin, acetylcholine, and motilin with inhibitors) were analyzed using one-way ANOVA (Brown-Forsythe and Welch ANOVA tests for non-matched and paired data, and Dunnett T3 multiple comparisons test for comparisons between the treatment groups and the motilin or control group), where *n* represents the number of repetitions. LGA tissues from three dogs were pooled in each experiment. Values of *p* less than 0.05 in the *t*-test or ANOVA were considered statistically significant.

## Results

### Motilin Induces Concentration-Dependent Relaxation of LGA Rings

**Figure 1:**
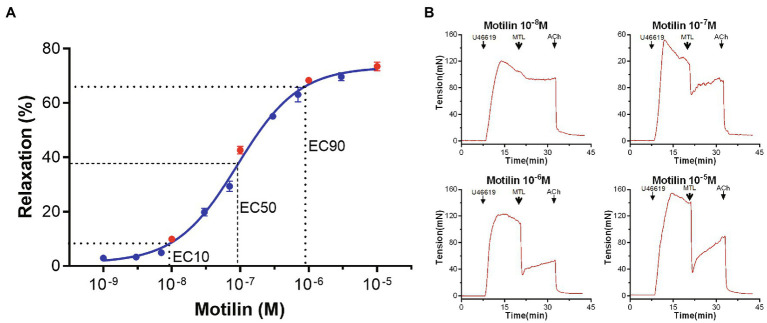
Concentration-response curve of motilin-induced relaxation of LGA and representative traces. **(A)** Motilin induces LGA relaxation in dogs in a concentration-dependent manner. Values are means±SEM (*n*=7). The dotted line shows the EC_10_, EC_50_, and EC_90_ on the concentration-response curve. **(B)** Motilin concentrations in the traces correspond to the red dots in panel **(A)**. The arrows indicate the time of drug administration. ACh, acetylcholine; EC, effective concentration; LGA, left gastric artery; MTL, motilin; and SEM, standard error of the mean; U46619, thromboxane A_2_ receptor agonist.

The concentration-response curve of motilin (10^−9^–10^−5^M) showed a classic inverted “S” shape on a semi-logarithmic plot (*n*=7; Figure 1; Supplementary Table 1). The EC_10_, EC_50_, and EC_90_ were 9.4×10^−9^±2.9×10^−9^M, 9.1×10^−8^±1.2×10^−8^M, and 8.8×10^−7^±3.4×10^−7^M, respectively, and the Hill slope was 1.0±0.1. These results indicate that motilin induces relaxation of LGA rings precontracted by U46619 in a concentration-dependent manner in the range from 1×10^−8^ to 7×10^−7^M. The EC_50_ of motilin (9×10^−8^M) was used in subsequent experiments.

### The Roles of the MLNR and Its Signal Transduction Pathway in LGA Relaxation

#### Role of the MLNR in Motilin-Induced Vasodilation

**Figure 2:**
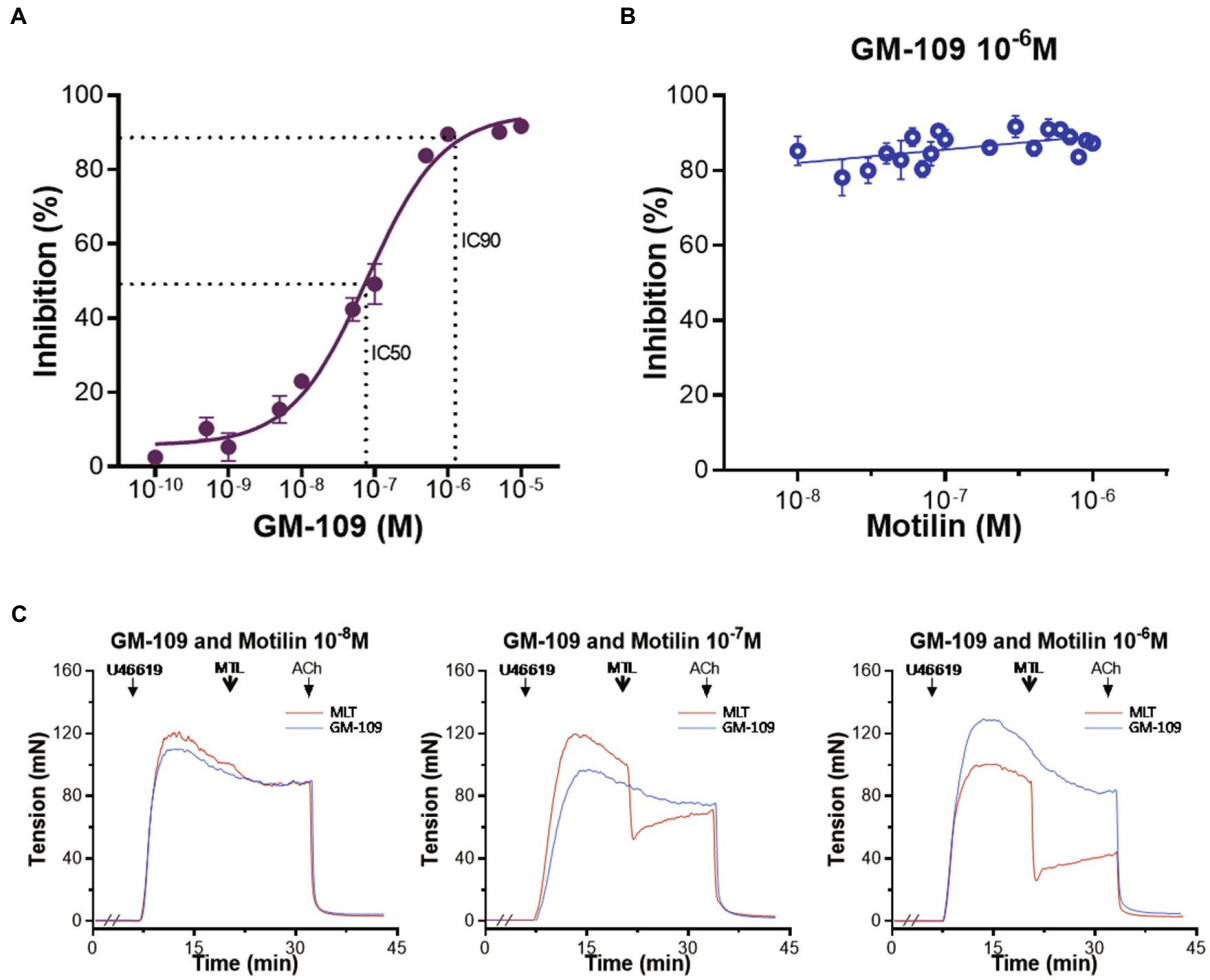
Role of the MLNR on motilin-induced relaxation of the LGA in dogs. **(A)** Concentration-response curve of the inhibition of motilin-induced relaxation of LGA rings by GM-109. Data are means±SEM (*n*=7). The motilin concentration used in the assay was 9×10^−8^M. The dotted line shows IC_50_ and IC_90_ on the concentration-response curve. **(B)** Scatter plots of the inhibitory effect of GM-109 (10^−6^M) on motilin-induced vasorelaxation. The motilin concentration used in the assay was 10^−8^–10^−6^M. Data are means±SEM (*n*=7). **(C)** Representative traces of motilin (10^−8^, 10^−7^, and 10^−6^M) before (red) and after (blue) incubation with GM-109 (10^−6^M). The double slash (//) indicates that GM-109 was added to the inhibitor group before treatment with U46619; arrows indicate the time of drug administration. ACh, acetylcholine; GM-109, MLNR antagonist; IC, inhibitory concentration; LGA, left gastric artery; MLNR, motilin receptor; MTL, motilin; SEM, standard error of the mean; and U46619, thromboxane A_2_ receptor agonist.

The role of the MLNR on vasorelaxation was evaluated using the MLNR antagonist GM-109 (Tankanashi et al., 1995). The results showed that GM-109 (10^−10^–10^−5^M) inhibited LGA relaxation induced by motilin (9×10^−8^M) in a concentration-dependent manner, and its concentration-response curve showed an inverted “S” shape (Figure 2A). The IC_50_ was 7.8×10^−8^±0.6×10^−8^M, the IC_90_ was 1.1×10^−6^±0.2×10^−6^M, and the Hill slope was 0.8±0.1. The RRs of LGA induced by motilin (9×10^−8^M) before and after treatment with GM-109 were compared using a paired *t*-test (*n*=7; Supplementary Table 2). A higher concentration of GM-109 (10^−6^M, equivalent to IC_90_) significantly inhibited vasorelaxation, with IRs ranging from 77.7%±2.8 to 89.3%±2.1% (Figure 2B). The RRs before and after treatment with GM-109 (10^−6^M) were compared using a paired *t*-test (*n*=7; Supplementary Table 3). Representative traces are shown in Figure 2C.

#### Role of the G Protein-PLC-IP_3_ Pathway in Motilin-Induced Vasodilation

**Figure 3:**
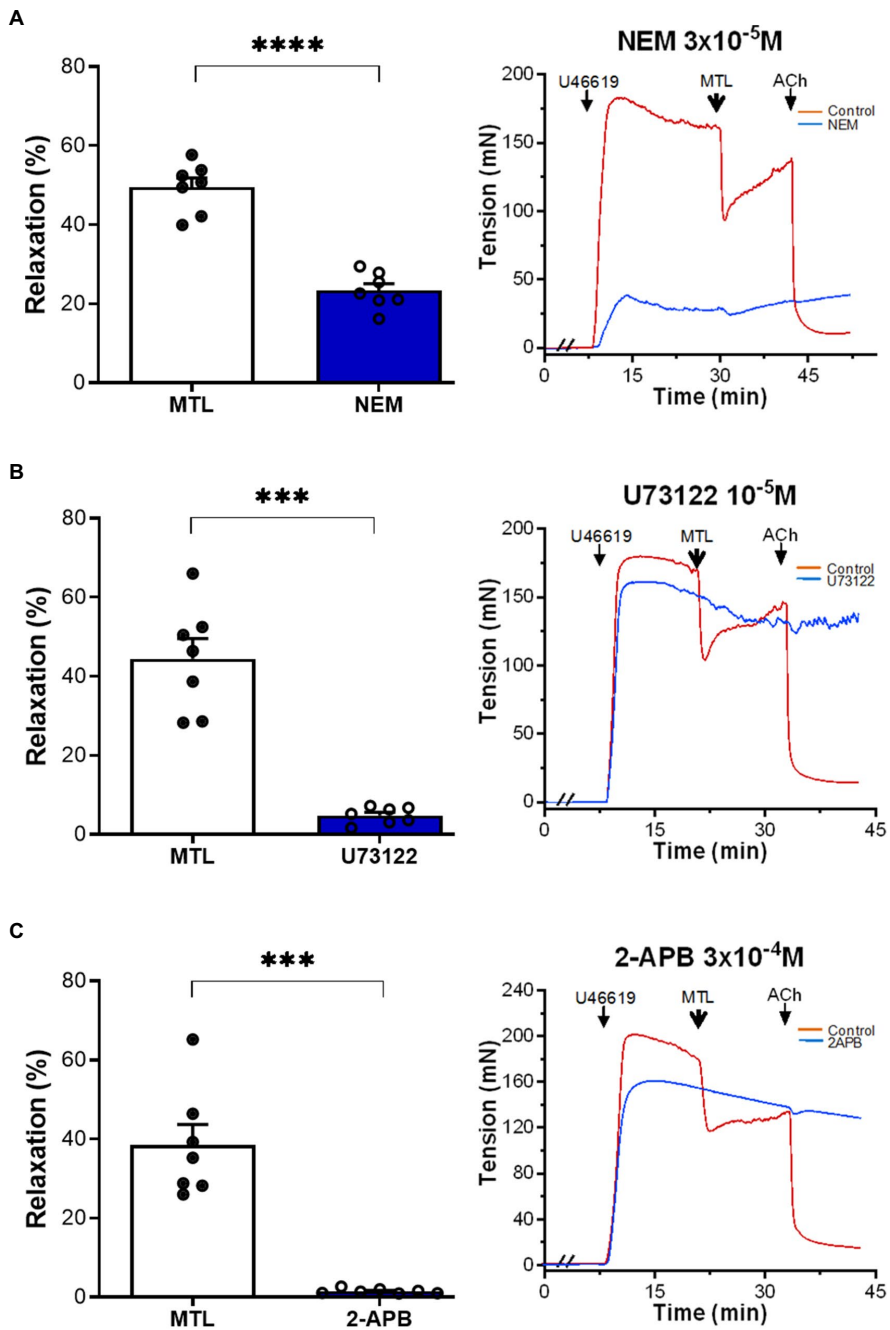
Role of the G protein-PLC-IP_3_ pathway on motilin-induced vasorelaxation. **(A–C)** Effect of NEM (3×10^−5^M), U73122 (10^−5^M), and 2-APB (3×10^−4^M) on LGA relaxation induced by motilin (9×10^−8^M) and corresponding representative traces. Values are means±SEM (*n*=7). ^***^p<0.001; ^****^p<0.0001 by paired *t*-test. The double slash (//) indicates pretreatment with NEM, U73122, or 2-APB before incubation with U46619. The arrows indicate the time of drug administration. 2-APB, 2-aminoethyl diphenylborinate (IP_3_ blocker); ACh, acetylcholine; IP_3_, inositol trisphosphate; LGA, left gastric artery; MTL, motilin; NEM, N-ethylmaleimide (G-protein antagonist); PLC, phospholipase C; SEM, standard error of the mean; U73122, PLC inhibitor; and U46619, thromboxane A_2_ receptor agonist.

The G protein antagonist N-ethylmaleimide (NEM) decreased the RR of LGA by 52.3±4.0%, reducing the RR from 49.5±2.4 to 23.4±1.7% (*n*=7, *p*<0.0001; Figure 3A). The PLC inhibitor U73122 reduced the RR by 88.5%±2.0%, reducing the RR from 44.4±5.2 to 4.9±0.8% (*n*=7, *p*=0.0002; Figure 3B). The IP_3_ receptor blocker 2-APB decreased vasodilation by 95.4±1.1%, reducing the RR from 38.5±5.2 to 1.6±0.2% (*n*=7, *p*=0.0004; Figure 3C).

#### Role of the Diacylglycerol-PKC Pathway on Motilin-Induced Vasorelaxation

**Figure 4:**
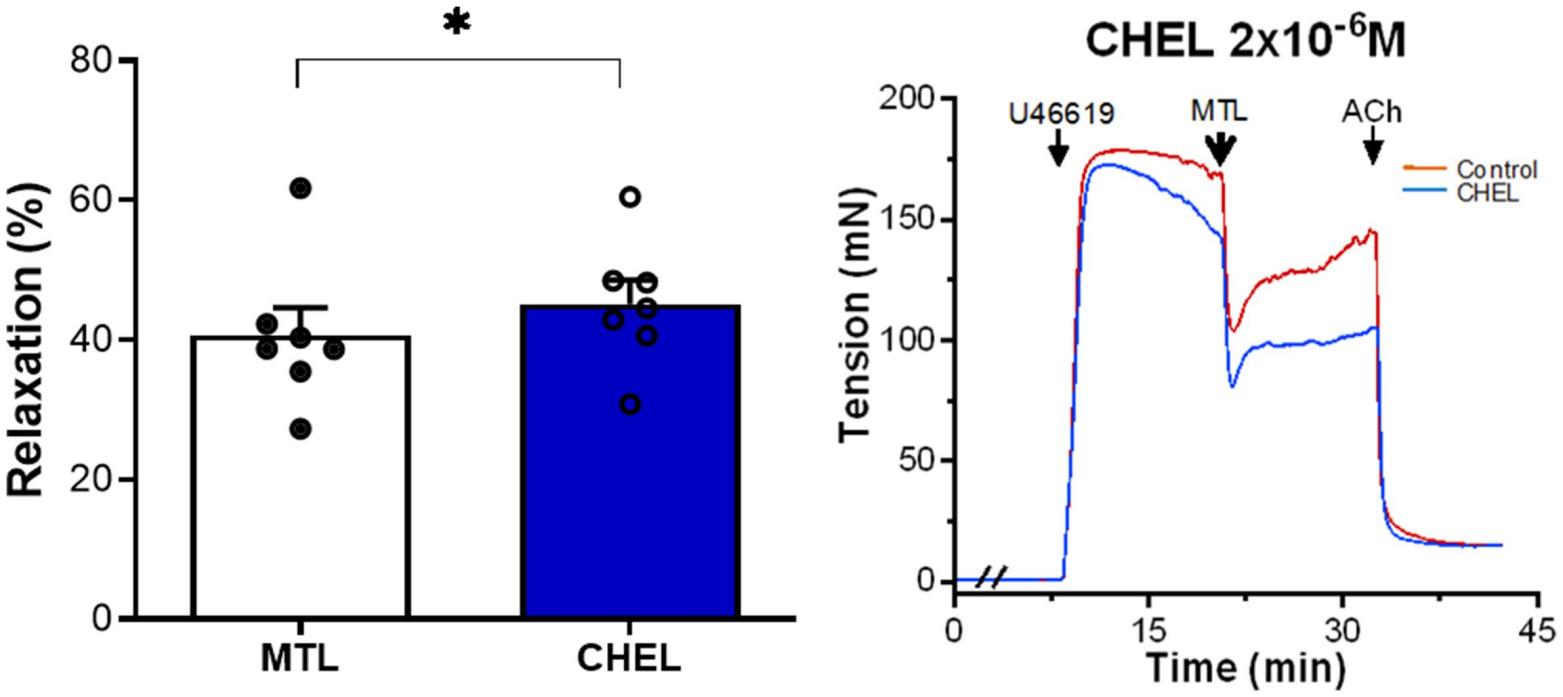
Role of the DG-PKC pathway on motilin-induced vasorelaxation. Effect of chelerythrine (2×10^−6^M) on LGA relaxation induced by motilin (9×10^−8^M) and corresponding representative traces. Values are means±SEM (*n*=7). ^*^p<0.05 by paired *t*-test. The double slash (//) indicates pretreatment with chelerythrine before incubation with U46619. The arrows indicate the time of drug administration. ACh, acetylcholine; CHEL, chelerythrine; DG, diacylglycerol; LGA, left gastric artery; MTL, motilin; PKC, protein kinase C; SEM, standard error of the mean; and U46619, thromboxane A_2_ receptor agonist.

The PKC inhibitor chelerythrine increased the RR from 34.9±2.9 to 39.7±3.7% with significance (*n*=7, *p*=0.012; Figure 4), showing no inhibitory effect on motilin-induced vasorelaxation.

#### Role of the Adenylate Cyclase-PKA Pathway in Motilin-Induced Vasorelaxation

**Figure 5:**
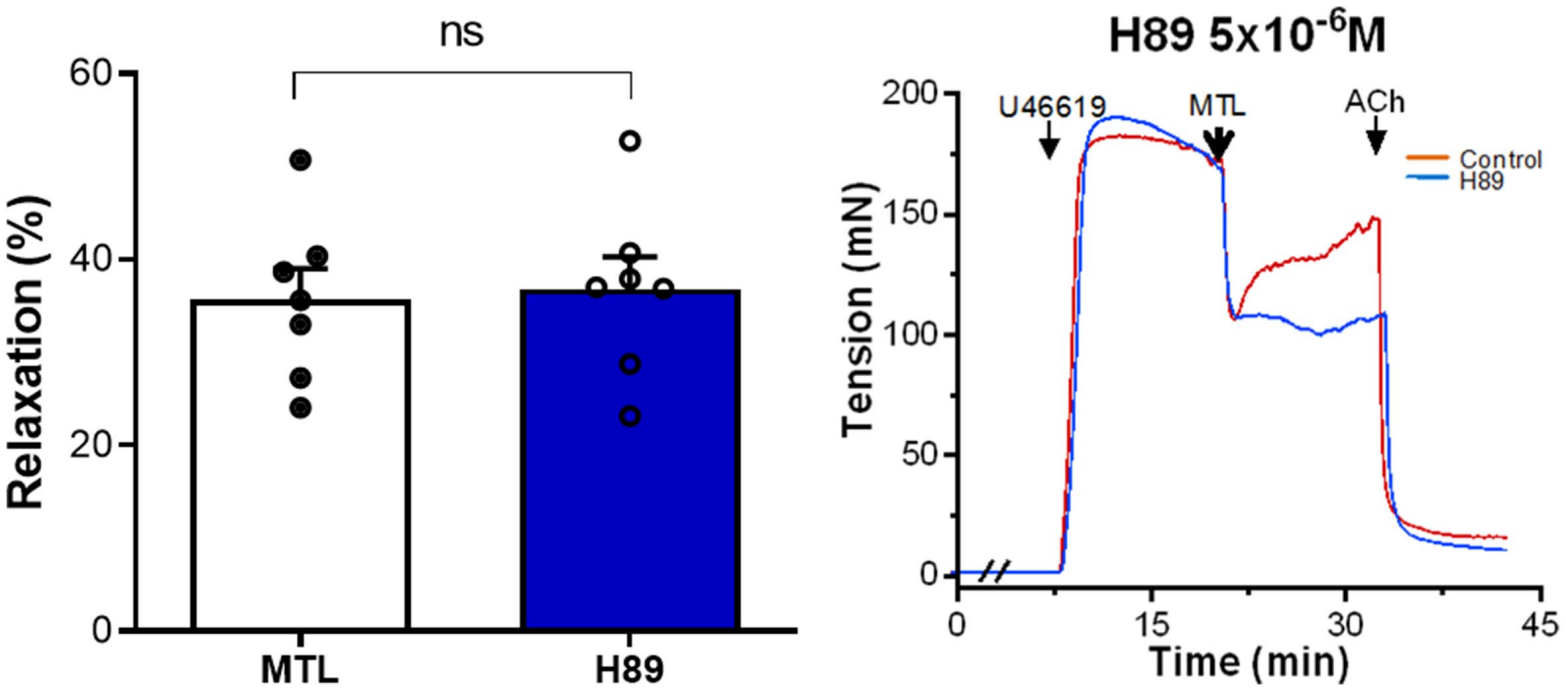
Role of the AC-PKA pathway on motilin-induced vasorelaxation. Effect of H89 (5×10^−6^M) on LGA relaxation induced by motilin (9×10^−8^M) and corresponding representative traces. Values are means±SEM (*n*=7). ns, non-significant at *p*>0.05 by paired *t*-test. The double slash (//) indicates pretreatment with H89 before incubation with U46619. The arrows indicate the time of drug administration. AC, adenylyl cyclase; ACh, acetylcholine; H89, PKA inhibitor; LGA, left gastric artery; MTL, motilin; PKA, protein kinase A; SEM, standard error of the mean; and U46619, thromboxane A_2_ receptor agonist.

The PKA inhibitor H89 increased the RR from 34.9±2.9 to 39.7±3.7% without significance (*n*=7, *p*=0.188; Figure 5).

### Effects of Extracellular [Ca^2+^] on Vasorelaxation

**Figure 6:**
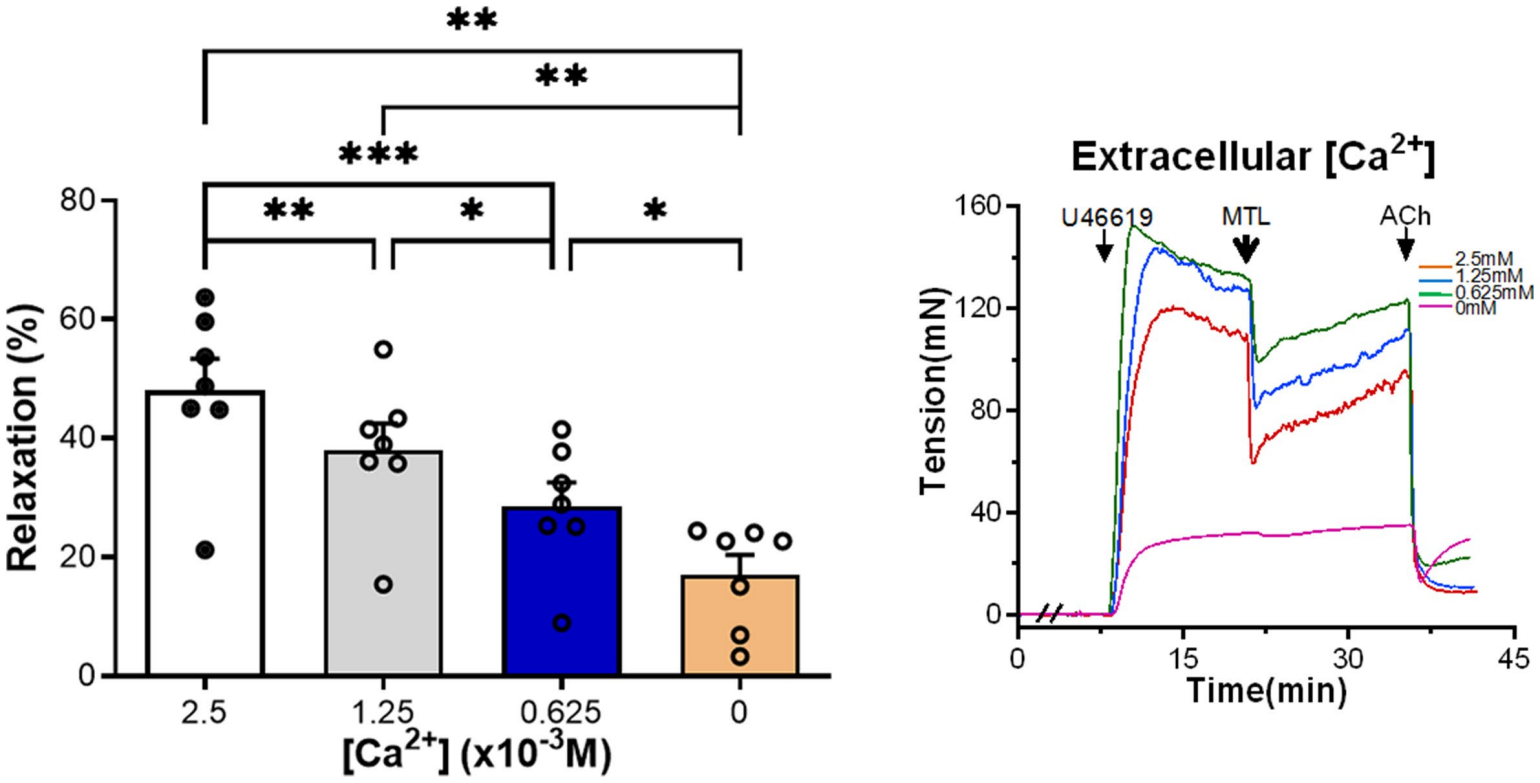
Role of extracellular [Ca^2+^] on motilin-induced vasorelaxation. The Krebs solutions containing different Ca^2+^ concentrations decreased motilin-induced LGA relaxation in a concentration-dependent manner. Data are means±SEM (*n*=7). ^*^p<0.05; ^**^p<0.01; and ^***^p<0.001 by one-way ANOVA. In representative traces, the double slash (//) indicates pretreatment with different Ca^2+^ concentrations before incubation with U46619. The arrows indicate the time of drug administration. ACh, acetylcholine; ANOVA, analysis of variance; LGA, left gastric artery; MTL, motilin; SEM, standard error of the mean; and U46619, thromboxane A_2_ receptor agonist.

Left gastric artery rings were incubated with Krebs solution containing [Ca^2+^] of 2.5×10^−3^M (control group), 1.25×10^−3^, 0.625×10^−3^, and 0M. As the [Ca^2+^] decreased, motilin-induced RRs also gradually reduced (*n*=7; Figure 6; Supplementary Table 4).

### Roles of Endothelium-Derived Relaxation Factors in Motilin-Induced Vasorelaxation

#### Role of the NOS–NO–sGC–cGMP Pathway in Vasorelaxation

**Figure 7:**
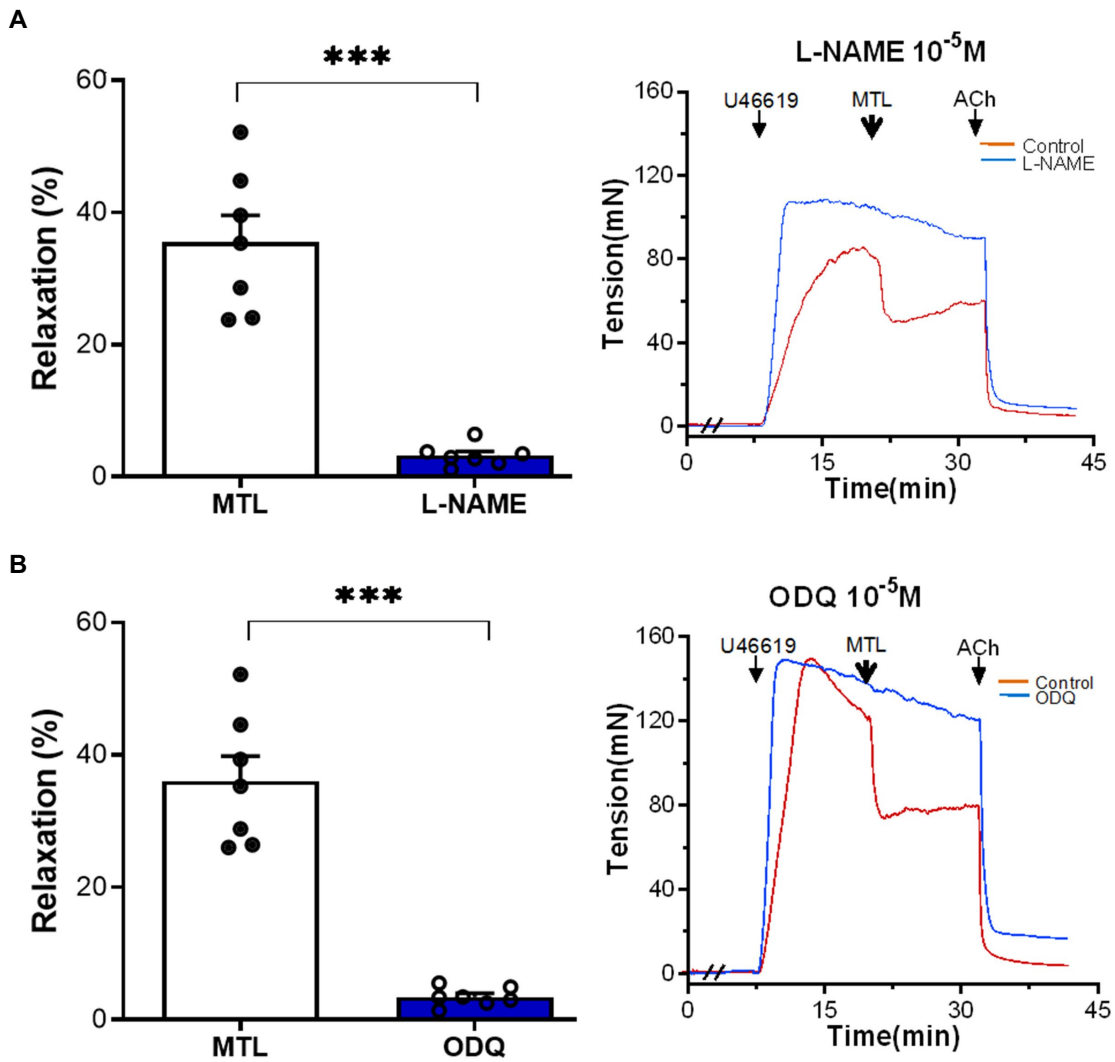
Role of the NOS–NO–sGC–cGMP pathway on motilin-induced vasorelaxation. **(A,B)** Effects of L-NAME (10^−4^M) and ODQ (10^−5^M) on LGA relaxation induced by motilin (9×10^−8^M) and corresponding representative traces. Values are means±SEM (*n*=7). ^***^p<0.001 by paired *t*-test. The double slash (//) indicates pretreatment with L-NAME and ODQ before incubation with U46619. The arrows indicate the time of drug administration. ACh, acetylcholine; cGMP, cyclic guanosine monophosphate; LGA, left gastric artery; L-NAME, N-nitro-L-arginine methyl ester (NOS inhibitor); MTL, motilin; NO, nitric oxide; NOS, nitric oxide synthase; ODQ, sGC inhibitor; SEM, standard error of the mean; sGC, soluble guanylyl cyclase; and U46619, thromboxane A_2_ receptor agonist.

The NOS inhibitor L-NAME decreased the RR of the LGA by 90.8±4.0% (from 35.5±4.1 to 3.2±0.6%; *n*=7, *p*=0.0001; Figure 7A). Similarly, the sGC inhibitor ODQ reduced RR by 90.2±1.6% (from 36.1±3.8 to 3.4±0.5%; *n*=7, *p*=0.0001; Figure 7B).

#### Role of PGI_2_ on Motilin-Induced Vasorelaxation

**Figure 8:**
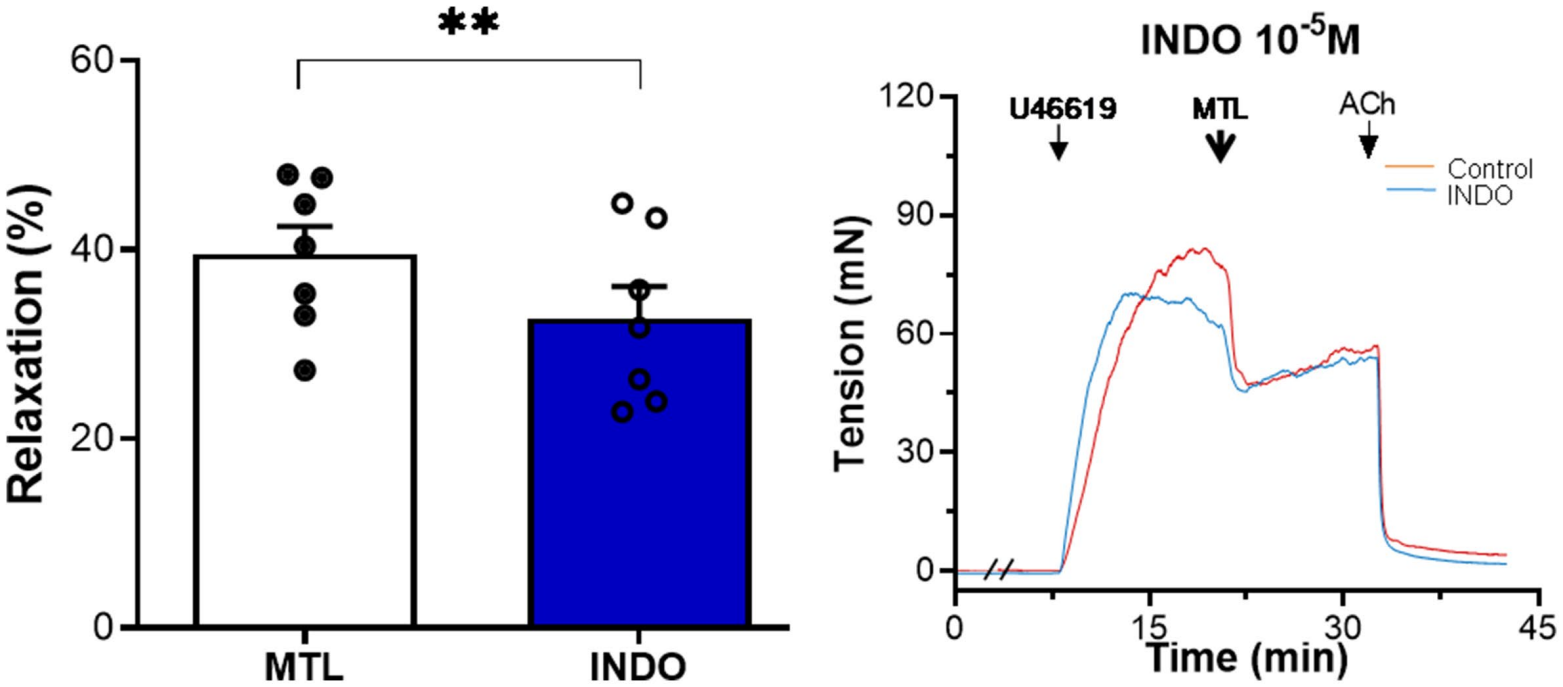
Role of PGI_2_ on motilin-induced vasorelaxation. Effect of indomethacin (10^−5^M) on LGA relaxation induced by motilin (9×10^−8^M) and corresponding representative trace. Values are means±SEM (*n*=7). ^**^p<0.01 by paired *t*-test. The double slash (//) indicates pretreatment with indomethacin before incubation with U46619. The arrows indicate the time of drug administration. INDO, indomethacin; LGA, left gastric artery; MTL, motilin; PGI_2_, prostacyclin; SEM, standard error of the mean; and U46619, thromboxane A_2_ receptor agonist.

The cyclooxygenase inhibitor indomethacin reduced vasodilation by 17.9±3.7%, reducing the RR from 39.5±3.0 to 32.7±3.4% (*n*=7, *p*=0.004; Figure 8).

#### Roles of MEGJ and K^+^ Channels on Motilin-Induced Vasorelaxation

**Figure 9:**
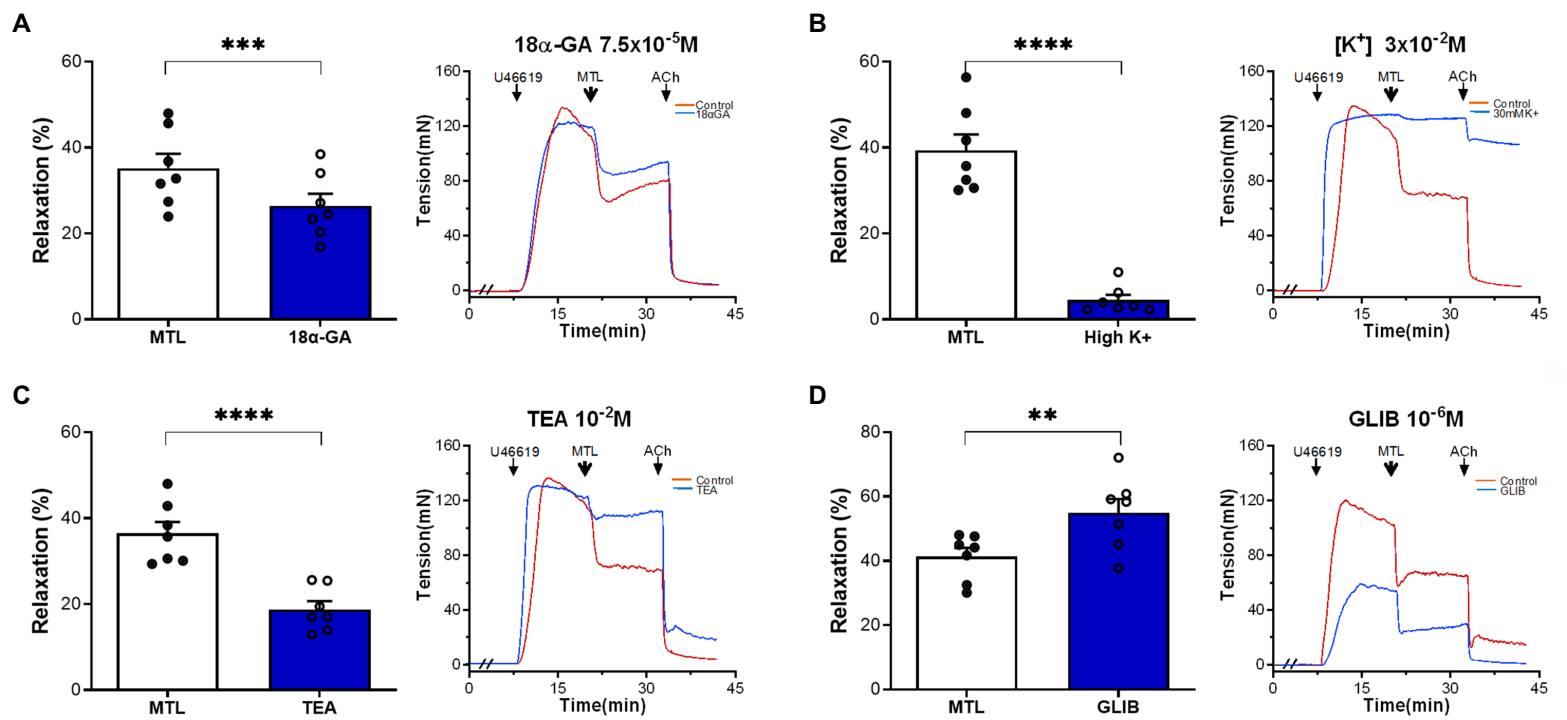
Roles of MEGJs and K^+^ channels on motilin-induced vasorelaxation. **(A–D)** Effects of 18α-GA (7.5×10^−5^M), high K^+^ solution (3×10^−2^M), TEA-Cl (10^−2^M), and glibenclamide (10^−6^M) on LGA relaxation induced by motilin (9×10^−8^M) and corresponding representative traces. Values are means±SEM (*n*=7). ^**^p<0.01; ^***^p<0.001; and ^****^p<0.0001 by paired *t*-test. The double slash (//) indicates 18α-GA, high K^+^ solution, TEA-Cl, or glibenclamide pretreatment before incubation with U46619. The arrows indicate the time of drug administration. 18α-GA, 18α-glycyrrhetinic acid (MEGJ inhibitor); ACh, acetylcholine; GLIB, glibenclamide (K_ATP_ channel blocker); LGA, left gastric artery; MEGJ, myoendothelial gap junction; MTL, motilin; SEM, standard error of the mean; TEA-Cl, tetraethylammonium chloride (K_Ca_ channel blocker); and U46619, thromboxane A_2_ receptor agonist.

The MEGJ inhibitor 18α-GA decreased vasorelaxation by 25.1±3.3%, reducing the RR from 35.2±3.4 to 26.4±2.9% (*n*=7, *p*=0.0006; Figure 9A).

A high [K^+^] solution containing 30mM KCl causes cell membrane depolarization through K^+^ channels (Nelson and Quayle, 1995). This solution decreased vasorelaxation by 87.9±2.1%, decreasing the RR from 42.4±4.4 to 5.5±1.4% (*n*=7, *p*<0.0001; Figure 9B). The non-specific K_Ca_ channel blocker TEA-Cl reduced LGA relaxation by 48.5±3.7%, reducing the RR from 36.5±2.7 to 18.8±1.9% (*n*=7, *p*<0.0001; Figure 9C). The K_ATP_ channel blocker glibenclamide increased the RR from 41.3±2.7 to 55.0±4.3% (*n*=7, *p*=0.002; Figure 9D), showing no inhibitory effect.

### Roles of the Endothelial MLNR and the NOS-NO-sGC-cGMP Pathway in Motilin-Induced Production of NO and cGMP

#### Motilin and Acetylcholine Stimulate the Production of NO and cGMP in LGA Tissues

**Figure 10:**
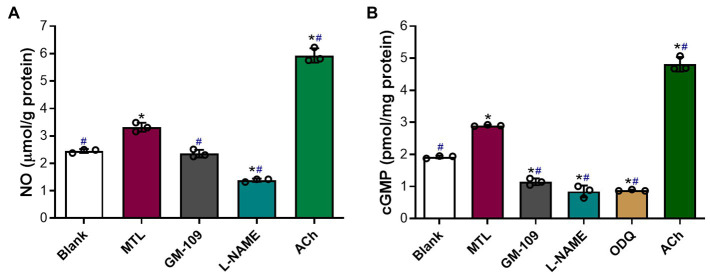
Roles of the MLNR and its signal transduction pathway in the motilin-induced production of NO and cGMP in LGA tissues. **(A,B)** Effect of agonists and inhibitors on the production of NO and cGMP in the LGA. Data are means±SEM (*n*=3). ^*^p< 0.05 vs. blank group; ^#^*p*<0.05 vs. motilin group by one-way ANOVA. The following drug concentrations were used: motilin, 9×10^−8^M; ACh, 10^−5^M; GM-109, 10^−5^M; L-NAME, 10^−4^M; and ODQ, 10^−5^M. ACh, acetylcholine; ANOVA, analysis of variance; cGMP, cyclic guanosine monophosphate; GM-109, MLNR antagonist; L-NAME, N-nitro-L-arginine methyl ester (NOS inhibitor); LGA, left gastric artery; MLNR, motolin receptor; MTL, motilin; NO, nitric oxide; ODQ, sGC inhibitor; and SEM, standard error of the mean.

The baseline levels of NO and cGMP in the blank group were 2.5±0.4μmolg^−1^ protein and 1.9±0.02pmolmg^−1^ protein, respectively (Figures 10A,B). Motilin increased the levels of NO and cGMP in LGA tissues by 1.3 and 1.5 times, respectively (*p*=0.0106 and *p*=0.0001). Acetylcholine (10^−5^M) increased the levels of NO and cGMP by approximately 2.4 and 2.5 times compared with baseline, respectively (*p*=0.0049 and *p*=0.0058), and approximately 1.8 and 1.7 times relative to motilin treatment, respectively (*p*=0.0020 and *p*=0.0129).

#### Roles of MLNR and NOS in Motilin-Induced Synthesis of NO and Roles of MLNR, NOS, and sGC in Motilin-Induced Synthesis of cGMP

GM-109 (10^−5^M) attenuated NO production in LGA tissues (*p*=0.0050) and markedly decreased the synthesis of cGMP (*p*=0.0041 vs. blank group and *p*=0.0034 vs. motilin group; Figures 10A,B).

L-NAME (10^−4^M) significantly decreased NO and cGMP levels (*p*=0.0037 and *p*=0.0279 vs. the blank group, and *p*=0.0063 and *p*=0.0076 vs. the motilin group, respectively).

The effect of ODQ (10^−5^M) on cGMP levels was similar to that of L-NAME (*p*=0.0001 vs. blank group and *p*<0.0001 vs. the motilin group).

## Discussion

**Figure 11:**
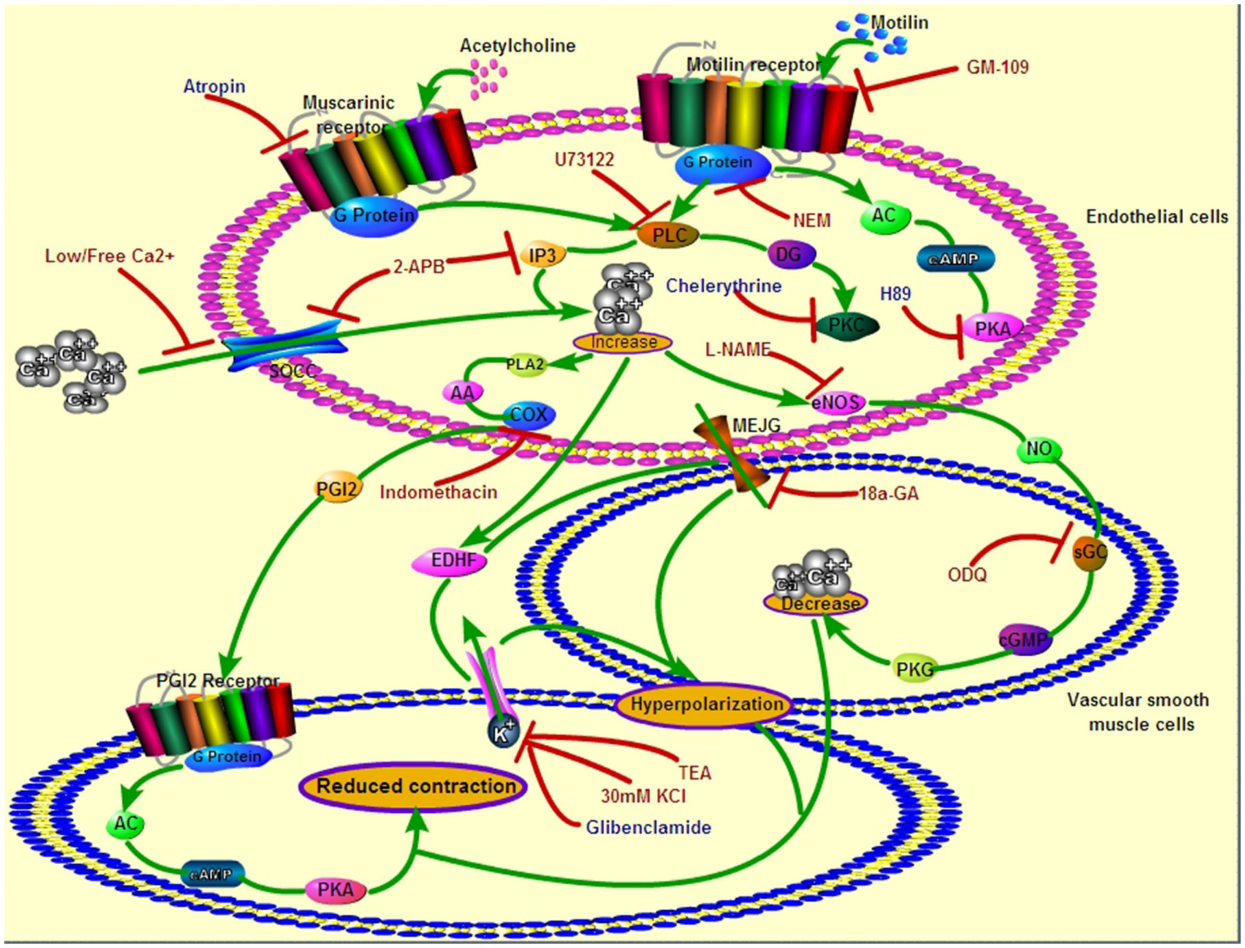
Roles of endothelial MLNR and its signal transduction pathway on LGA SMC relaxation. Green arrows indicate the signaling pathways in EC and SMC during motilin- or acetylcholine-induced relaxation of the dog LGA. Red arrows with flat heads indicate the blocking effect of inhibitors on the signaling pathways. The drugs in red indicate an inhibitory effect on MLNR signaling pathways, and those in blue indicate no inhibitory effect. EC, endothelial cell; LGA, left gastric artery; MLNR, motolin receptor; and SMC, smooth muscle cell.

Recent studies have shown that MLNRs are present on the ECs of gastrointestinal arteries (Yang et al., 2021), and endothelial MLNR is the molecular basis for the regulation gastric blood flow by motilin in dogs (Jin et al., 2002). The present study identified endothelial MLNR signaling pathways that induce VSM relaxation in the LGA of dogs (Figure 11).

Phase III of the migrating motor complex is marked by peristaltic waves of electrical activity that propagate from the lower esophagus along the gastrointestinal tract to clear excessive bacteria and Luminal contents (Deloose et al., 2012; Takahashi, 2013). Motilin from pigs and dogs has been shown to induce MMC III in dogs to a similar extent (Poitras et al., 1983), and injections with porcine motilin (at 12.5, 25, 50, and 100pmol/kg/h) simultaneously induces gastric MMC III and a sustained increase in LGA blood flow (Jin et al., 2002). Thus, porcine motilin was used for the assays in the current study. MLNR is differentially expressed in gastrointestinal arteries, with preferential expression and the highest motilin-induced relaxation in the LGA (Yang et al., 2021). Under physiological conditions, motilin periodically increased the blood flow of the LGA; however, the blood flow in the superior mesenteric artery (SMA) remained unchanged (Jin et al., 2002). Therefore, the LGA was used to identify the signal transduction pathway by which MLNR induces VSM relaxation.

GM-109 is a selective and competitive antagonist of MLNR in rabbit duodenum SMCs (Tankanashi et al., 1995). Data from the current study reveal that GM-109 also has an effective inhibitory effect on endothelial MLNR. The results describe that motilin-induced LGA relaxation depends on GM-109-sensitive MLNR in greater detail than a previous study (Yang et al., 2021).

Upon activation, MLNR is coupled to the G_q_ protein (Depoortere and Peeters, 1995; Feighner et al., 1999). The inhibitory effects of NEM, U73122, and 2-APB confirm that motilin acts *via* the G protein-PLC-IP_3_ signal transduction pathway. Motilin has been shown to increase intracellular [Ca^2+^] in HEK-293/aeq17 cells transfected with MLNR *via* this same pathway (Feighner et al., 1999). In addition, both the motilin-dependent increase of intracellular [Ca^2+^] and rabbit gastrointestinal SMC contraction also signal through this pathway (Depoortere and Peeters, 1995; Huang et al., 2005). Diacylglycerol (DG) is another important second messenger in GPCR signaling under the action of PLC, which further leads to the activation of PKC. However, the non-inhibitory effect of chelerythrine excluded the potential contribution of the PLC-DG-PKC pathway. Adenylate cyclase (AC)-PKA is also an essential intracellular signal transduction pathway downstream of G proteins (Ghanemi, 2015); however, the role of AC-PKA was excluded by the non-inhibitory effect of H89 in motilin-induced vasorelaxation. In line with these results, the intracellular signaling cascades involved in motilin-induced gastrointestinal smooth muscle contraction in rabbits do not depend on PKA (Depoortere and Peeters, 1995). Thus, the MLNR-G protein-PLC-IP_3_ signal transduction pathway is shared between ECs and gastrointestinal SMCs.

It was shown that the initial increase in cytosolic [Ca^2+^] in ECs of porcine aortic valves was due to Ca^2+^ release from intracellular stores, whereas the maintenance of a stable [Ca^2+^] was associated with Ca^2+^ influx (Higuchi et al., 1994). It was hypothesized that the motilin-activated MLNR-G protein-PLC-IP_3_ pathway induced Ca^2+^ release from the endoplasmic reticulum (ER) in ECs (Neves et al., 2002). Importantly, a reduction in extracellular [Ca^2+^] resulted in a synchronous decrease in vasorelaxation, indicating that extracellular Ca^2+^ is involved in motilin-induced LGA relaxation. Extracellular Ca^2+^ also participates in the motilin-induced contraction of SMCs (Kato et al., 2019). However, Ca^2+^ enters excitable SMCs and non-excitable ECs mainly *via* voltage-operated Ca^2+^ channels and store-operated Ca^2+^ channels (SOCCs), respectively (Tsoukias, 2011). SOCCs are controlled by Ca^2+^ stores in the ER (Groschner et al., 2017). The inhibitory effect of 2-APB further confirms the role of SOCCs in motilin-induced vasorelaxation.

The increase in cytosolic [Ca^2+^] induces the secretion of vasorelaxant substances from ECs (Lopez-Jaramillo et al., 1990). The inhibitory effects of L-NAME and ODQ indicate that the NOS–NO–sGC-cGMP pathway plays a crucial role. PGI_2_ also participated in the process; however, the low inhibitory efficacy of indomethacin suggests a negligible contribution of PGI_2_ (Hiroaki et al., 1996). EDHF increases cytosolic [Ca^2+^], which opens Ca^2+^-activated K^+^ channels and hyperpolarizes ECs. Next, direct electrical coupling through myoendothelial gap junctions (MEGJs) and K^+^ accumulation in the intercellular space induces endothelium-dependent hyperpolarization (EDH) of SMCs, leading to VSM relaxation (Félétou, 2016). The fact that 18α-GA and high K^+^ solution inhibited vasodilation, indicating the involvement of EDHF in motilin-induced vasorelaxation. Furthermore, it is likely that the K_Ca_ channel, but not the K_ATP_ channel, plays a role. This study is the first to report the effects of three endothelial-derived relaxation mediators in MLNR-dependent VSM relaxation in the LGA.

Motilin receptor agonists used in the treatment of diabetic gastroparesis improve delayed gastric emptying and mimic gastric MMC III (Barshop and Kuo, 2015; Sanger and Furness, 2016; Kumar et al., 2018; Zhong et al., 2020). Gastric blood supply is lower in patients with diabetic gastroparesis compared to healthy subjects (Shen et al., 2016). The decrease in microvascular perfusion may lead to neuropathy (Tomesˇova´ et al., 2013; Wang et al., 2015), which in turn decreases gastric motility (Kumar et al., 2018). The decrease in NO production or release is the primary manifestation of endothelial dysfunction in diabetic microangiopathy (Zhang et al., 2018). The current study verified that NO was critical for MLNR-dependent VSM relaxation in the LGA. These results suggest that the effects of motilin on gastric blood flow (Jin et al., 2002) are related to its regulation of the digestive tract-brain-pancreatic axis (Singaram et al., 2020). In this respect, the molecular mechanism by which the endothelial-derived MLNR induces VSM relaxation in the LGA may help elucidate the pathogenesis of diabetic gastroparesis and improve the prevention and treatment of this gastric complication.

In summary, motilin induces VSM relaxation in the LGA mainly *via* the endothelial MLNR-Gpr-PLC-IP_3_ and NOS-NO-sGC-cGMP signaling pathways. Extracellular Ca^2+^, PGI_2_, and EDHF are also involved in this process. These pathways constitute the molecular mechanism by which motilin regulates LGA blood flow under physiological conditions, and these data may serve as the basis for understanding and treating gastric diseases.

## Data Availability Statement

The original contributions presented in the study are included in the article/Supplementary Material, further inquiries can be directed to the corresponding author.

## Ethics Statement

The animal study was reviewed and approved by Animal Care and Use Committee of Jilin University (Permit No. 2016301).

## Author Contributions

HL: data curation and analysis, statistical analysis, and manuscript writing. LY: data curation and analysis and manuscript revision for important intellectual content. YJ: project supervision and data analysis and validation. CJ: study conceptualization, project administration, and funding acquisition. All authors contributed to the article and approved the submitted version.

## Funding

This work was supported by the National Natural Science Foundation of China (Grant No. 31271235, received by CJ).

## Conflict of Interest

The authors declare that the research was conducted in the absence of any commercial or financial relationships that could be construed as a potential conflict of interest.

## Publisher’s Note

All claims expressed in this article are solely those of the authors and do not necessarily represent those of their affiliated organizations, or those of the publisher, the editors and the reviewers. Any product that may be evaluated in this article, or claim that may be made by its manufacturer, is not guaranteed or endorsed by the publisher.

## Figures and Tables

**Table:** 

ACh	Acetylcholine
AC	Adenylate cyclase
ANOVA	Analysis of variance
cGMP	Cyclic guanosine monophosphate
DG	Diacylglycerol
DMSO	Dimethylsulfoxide
EC	Endothelial cell
EDH	Endothelium-dependent hyperpolarization
EDHF	Endothelium-dependent hyperpolarization factor
EGTA	Ethylene glycol tetra-acetic acid
ER	Endoplasmic reticulum
GPCR	G protein-coupled receptor
IP_3_	Inositol trisphosphate
IR	Inhibition rate
L-NAME	N-nitro-L-arginine methyl ester
LGA	Left gastric artery
MEGJs	Myoendothelial gap junctions
MLNR	Motilin receptor
MMC III	Phase III of the migrating motor complex
NEM	N-ethylmaleimide
NO	Nitric oxide
NOS	Nitric oxide synthase
PGI_2_	Prostacyclin
PKA	Protein kinase A
PKC	Protein kinase C
PLC	Phospholipase C
RR	Relaxation rate
sGC	Soluble guanylyl cyclase
SMA	Superior mesenteric artery
SMC	Smooth muscle cells
SOCC	Store-operated Ca^2+^ channel
TEA-Cl	Tetraethylammonium chloride
VSM	Vascular smooth muscle
2-APB	2-aminoethyl diphenylborinate
18α-GA	18α-glycyrrhetinic acid
