# Supplementary material for: Roles of Endothelial Motilin Receptor and Its Signal Transduction Pathway in Motilin-Induced Left Gastric Artery Relaxation in Dogs
Source: Front Physiol. 2021 Oct 28;12:770430. doi: 10.3389/fphys.2021.770430 (PMC8581264; doi:10.3389/fphys.2021.770430)
Supplement: Supplementary file 1 [file Data_Sheet_1.pdf]

Supplementary Materials

Supplementary Table 1. Relaxation rates of the LGA induced by different concentrations of motilin.

Concentration (M)	Relaxation rate (%)	vs. before value P-value
1×10^{-9}	2.9 ± 0.4	--
3×10^{-9}	3.2 ± 0.3	> 0.9999
7×10^{-9}	4.9 ± 0.9	0.9556
1×10^{-8}	9.9 ± 0.6*	0.0463
3×10^{-8}	19.8 ± 1.4**	0.0084
7×10^{-8}	29.4 ± 1.9**	0.0025
1×10^{-7}	42.6 ± 1.4*	0.0328
3×10^{-7}	55.1 ± 1.0***	0.0008
7×10^{-7}	63.1 ± 2.7	0.4641
1×10^{-6}	68.4 ± 0.9	0.8967
3×10^{-6}	69.7 ± 1.5	> 0.9999
1×10^{-5}	73.5 ± 1.6	0.9576

The experiments were conducted as described in Figure 1. *P < 0.05; **P < 0.01; and ***P < 0.001 by one-way ANOVA. Values are means ± SEM (n = 7).

Supplementary Table 2. Relaxation rates of the LGA induced by motilin (9×10^{-8} M) before and after treatment with GM-109 (10^{-10} to 10^{-5} M).

Concentration of GM-109 (M)	Relaxation rate in control (%)	Relaxation rate after treatment with GM-109 (%)	P-value	Inhibition rate (%)
1×10^{-10}	42.0 ± 4.1	40.9 ± 4.1	0.2209	2.6 ± 1.7
5×10^{-10}	41.6 ± 3.9	37.5 ± 3.9*	0.0229	10.3 ± 3.0
1×10^{-9}	40.1 ± 5.0	37.5 ± 4.2	0.1121	5.3 ± 3.8
5×10^{-9}	37.0 ± 3.5	31.2 ± 3.3**	0.0075	15.5 ± 3.7
1×10^{-8}	38.0 ± 2.9	29.2 ± 2.2***	0.0002	23.0 ± 1.8
5×10^{-8}	34.9 ± 2.4	20.2 ± 2.1****	< 0.0001	42.4 ± 3.1
1×10^{-7}	36.2 ± 3.8	19.3 ± 3.5****	< 0.0001	49.2 ± 5.5
5×10^{-7}	35.5 ± 3.3	5.7 ± 0.8****	< 0.0001	83.8 ± 2.0
1×10^{-6}	38.5 ± 1.6	3.9 ± 0.3****	< 0.0001	89.5 ± 1.0
5×10^{-6}	34.2 ± 4.6	3.2 ± 0.6***	0.0004	90.1 ± 1.9
1×10^{-5}	40.5 ± 2.1	3.3 ± 0.5****	< 0.0001	91.7 ± 1.4

The experiments were conducted as described in Figure 2A. *P < 0.05; **P < 0.01; ***P < 0.001; and ****P < 0.0001 by unpaired t-test. Values are means ± SEM (n = 7).

Supplementary Table 3. Relaxation rates of the LGA induced by motilin (10^{-8} to 10^{-6} M) before and after treatment with GM-109 (10^{-6} M).

Motilin concentration (M)	Relaxation rate in the control (%)	Relaxation rate upon treatment with GM-109 (%)	P-value	Inhibition rate (%)
1×10^{-8}	10.9 ± 1.2	2.3 ± 0.1**	0.0020	77.7 ± 2.8
2×10^{-8}	12.0 ± 1.4	2.4 ± 0.4**	0.0016	79.6 ± 2.8
3×10^{-8}	17.8 ± 2.3	2.9 ± 0.6**	0.0025	83.7 ± 3.8
4×10^{-8}	19.9 ± 0.6	3.0 ± 0.5****	<0.0001	84.5 ± 2.7
5×10^{-8}	21.7 ± 2.0	3.2 ± 0.9***	0.0007	85.0 ± 4.0
6×10^{-8}	23.8 ± 1.6	3.2 ± 0.4***	0.0001	86.4 ± 1.7
7×10^{-8}	25.2 ± 2,8	3.4 ± 0.3**	0.0018	85.6 ± 2.3
8×10^{-8}	28.4 ± 2.2	3.9 ± 0.9***	0.0002	86.5 ± 2.6
9×10^{-8}	33.8 ± 2.8	4.1 ± 0.6***	0.0006	87.1 ± 2.8
1×10^{-7}	37.6 ± 3.3	4.3 ± 0.8**	0.0010	87.8 ± 2.9
2×10^{-7}	42.5 ± 2.5	5.4 ± 0.8****	<0.0001	87.2 ± 1.7
3×10^{-7}	53.8 ± 2.9	6.0 ± 1.3****	<0.0001	89.3 ± 2.1
4×10^{-7}	49.4 ± 2.7	6.8 ± 0.8***	0.0002	86.0 ± 2.1
5×10^{-7}	53.8 ± 5.0	7.8 ± 1.2***	0.0006	85.4 ± 2.0
6×10^{-7}	60.5 ± 2.1	8.0 ± 1.3****	<0.0001	86.8 ± 2.1
7×10^{-7}	58.1 ± 4.8	8.4 ± 1.7**	0.0014	85.5 ± 4.9
8×10^{-7}	59.2 ± 4.8	9.4 ± 0.8***	0.0003	84.1 ± 0.7
9×10^{-7}	58.5 ± 2.5	9.1 ± 1.5***	0.0001	84.2 ± 2.9
1×10^{-6}	68.7 ± 2.4	9.7 ± 0.7****	<0.0001	85.9 ± 0.9

The experiments were conducted as described in Figure 2B. **P < 0.01; ***P < 0.001; and ****P < 0.0001 by unpaired t-test. Values are means ± SEM (n = 5).

Supplementary Table 4. Relaxation rates of the LGA induced by motilin (9 × 10^{-8} M) after treatment with Krebs solutions containing different [Ca^{2+}].

Extracellular [Ca^{2+}] (mM)	Motilin- induced relaxation rate (%)	P-value vs. 2.5 mM	P-value vs. 1.25 mM	P-value vs. 0.625 mM
2.5	48.1 ± 5.2	---	0.0086	0.0007
1.25	38.0 ± 4.5	0.0086	---	0.0266
0.625	28.0 ± 4.0	0.0007	0.0266	---
0	17.0 ± 3.3	0.0025	0.0061	0.0266

The experiments were conducted as described in Figure 6. Statistical significance was analyzed by one-way ANOVA. Values are means ± SEM (n = 7).
